# FMCW Laser Fuze Multiple Scattering Model and Accurate Fixed-Distance Algorithm in a Smoke Environment

**DOI:** 10.3390/s20092604

**Published:** 2020-05-03

**Authors:** Chengtian Song, Ying Cui, Bohu Liu

**Affiliations:** School of Mechatronical Engineering, Beijing Institute of Technology, Beijing 100081, China; songct@bit.edu.cn (C.S.); cuiyingvvv@163.com (Y.C.)

**Keywords:** FMCW laser fuze, multiple scattering, somke interference, CSD algorithm, accurate fixed-distance algorithm

## Abstract

In a smoke environment, suspended particles can scatter and absorb laser photons, making target echo signals extremely weak and difficult to extract and identify, which causes obvious difficulty in fixed-distance of laser fuze. In this paper, the multiple scattering model of frequency-modulated-continuous-wave (FMCW) laser fuze in a smoke environment was established. This model simulates multi-path propagation and multiple scattering of photons. At the same time, we use the correntropy spectral density (CSD) algorithm for accurate fixed-distance of FMCW laser fuze. The absolute error of distance does not exceed 0.15 m in smoke interference environment.

## 1. Introduction

Fixed-distance fuze is a device that measures the distance to the target by radio, laser, ultra-wideband, millimeter wave, and detonates the warhead at the best explosive point. Pulse laser fuze has been widely used for its characteristics of excellent anti-electromagnetic interference capability, simple structure, and low cost. However, pulse laser fuze is susceptible to interference from suspended particles such as smoke and clouds, which makes it difficult to extract and identify target signals [1,2]. Compared with pulse laser, the frequency modulated continuous wave (FMCW) laser ranging system depends on the beat frequency instead of the magnitude and time of the echo signal so that it has a natural advantage in target detection under dense fog conditions [3,4]. Therefore, it is very meaningful to improve the anti-interference ability of FMCW laser fuze in smoke environments.

Research has been done on the target echo characteristics of laser in the interference environment of suspended particles. Grabner et al. studied the effect of extinction coefficients of different concentrations of fog and haze on laser transmission [5]. Lundin et al. studied the transmission characteristics of FMCW laser signals in solid scattering media [6]. Zhang et al. studied the characteristics of smoke backscatter interference signals of FMCW laser fuze, and proposed a method for detecting intermediate frequency signals based on a normalized threshold [7,8]. In our earlier work, a Monte Carlo simulation model of FMCW laser transmission in a smoke interference environment was established [9]. The parameters of the aerosol scattering coefficient and extinction coefficient change randomly during the dynamic approach. In addition, some traditional denoising methods, including empirical mode decomposition (EMD) direct wavelet thresholding, EMD interval thresholding, correlation-based EMD partial reconstruction, discrete Fourier transform (DFT), and wavelet transformation, are investigated to provide a comparison with the cross-power-spectral-density (CPSD) algorithm [10]. Both the simulation and experiment results showed that the CPSD algorithm has better performance than other algorithms for the anti-interference of FMCW laser detection. However, the CPSD system is based on four photodetectors, and the complex and expensive disadvantages limit its application in FMCW laser fuze. This paper provides a new CSD algorithm for fixed-distance. In addition, the accuracy achieved by CPSD using four detectors can be carried out with only one detector.

Corretropy is used to compare the degree of similarity between the two sets of signals. It was first proposed by Jose C. Principe, a professor of electrical and computer engineering at the University of Florida, and analyzed in detail [11,12]. Garde et al. conducted systematic theoretical analysis, applied research on correntropy, and defined the Fourier transform of correntropy as the Corretropy Spectral Density (CSD) function [13,14,15,16,17]. The correntropy performs well in signal processing, image processing, artificial intelligence, etc. Chen et al. designed an adaptive filter based on the generalized maximum correntropy criterion to maximize the correlation between the input signal and the output signal, thereby effectively suppressing noise [18]. Yu et al. proposed a time delay estimation algorithm based on the weighted correntropy spectral density and this method was accurate in the presence of Gaussian or impulsive noise with a low generalized signal-to-noise ratio [19]. In view of the advantages of CSD algorithm in determining the similarity of two sets of signals, this paper chooses the CSD algorithm to process an intermediate frequency signal.

In this paper, we established a laser multipath propagation model in smoke interference environment in Section 2. The process of using the CSD algorithm to determine the distance was described in Section 3. In Section 4, the feasibility and performance of the CSD algorithm were evaluated and compared with FFT algorithm by MATLAB simulation (Matlab R2019a, MathWorks, Natick, MA, USA) in various environments. In Section 5, we established the system of fixed-distance of FMCW laser fuze and carried out the experiment. The experimental results were consistent with the simulation results. Finally, the conclusions are brought in Section 6.

## 2. FMCW Laser Multiple Scattering Model in the Smoke Interference Environment

**Figure 1:**
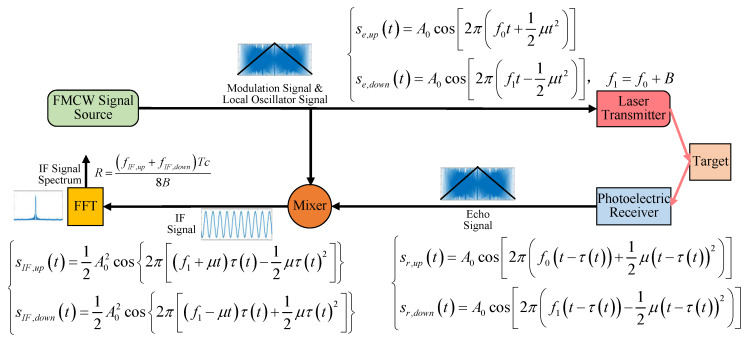
Frequency-modulated-continuous-wave (FMCW) laser detection principle. IF signal of FMCW laser fuze is obtained by mixing the echo signal with the local oscillator signal.

The FMCW laser ranging system modulates the light intensity of transmitted laser signal by a signal in which instantaneous frequency varies linearly with time and uses the modulation signal as a local oscillator signal. Due to the time delay of laser transmission in the atmosphere, the instantaneous frequency of the echo signal changes. The echo signal is mixed with the local oscillator signal to obtain the intermediate frequency (IF) signal. When there is no interference, the IF signal should be a cosine signal with a certain frequency. The frequency is related to the target distance and relative speed. Symmetric triangular wave FMCW modulation is widely used because it can directly calculate the distance and speed of the target from the IF signal. The detection principle is shown in Figure 1, where the initial phase of the modulation signal is zero, and the echo signal does not consider the phase shift and amplitude attenuation for the time being, where $f_{0}$ is the initial frequency, *c* is speed of light, $A_{0}$ is amplitude of the transmitted signal, τ is time delay between the echo signal and the transmitted signal, and μ is Frequency modulation slope. The calculation formula is μ=2B/T. *T* is modulation period and *B* is modulation bandwidth.

For the convenience of research, this model only analyzes the up-sweep period. At this time, the up-sweep modulation period $T_{m}=0.5T$ and the IF frequency of the up-sweep period satisfy $f_{IF}=f_{IF,up}=f_{IF,down}$. Then, there is:

(1) $$R=\frac{\left(f_{IF,up}+f_{IF,down}\right)Tc}{8B}=\frac{\left(f_{IF}+f_{IF}\right)2T_{m}c}{8B}=\frac{T_{m}c}{2B}f_{IF}$$

The target distance *R* need to be calculated by $f_{IF}$.

**Figure 2:**
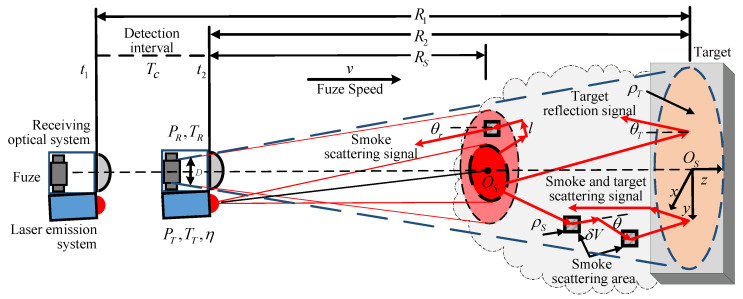
Multiple scattering model and detection process, simulating the movement of fuze with speed of *v* and a time interval of $T_{c}$ between two detections.

Existing simulations of FMCW laser fuzes in smoke interference environment assume that both the fuze and target are in a stable smoke environment, assuming that the smoke scattering coefficient and extinction coefficient are fixed values. In fact, as fuze gradually approaches the target covered by smoke, the occurrence of smoke interference is a random dynamic process from scratch. In our earlier work, the aerosol extinction coefficient and scattering coefficient changed dynamically according to the different smoke density variation, aerosol particle distribution, and photon spatial positions. Based on [9], in this paper, we take account of the multiple scattering and multipath propagation of photons, and establish a multiple scattering model of the FMCW laser in the smoke environment. The scattering model and detection process are shown in Figure 2.

According to the transmission process of the laser in the smoke environment, three types of echo signal photons can be received by the fuze receiving system at a certain time, including: (1) photons that are directly transmitted to the target surface after being emitted, and are directly received by the fuse after being reflected by the target; (2) photons that do not reach the target surface scattered by the aerosol and return to the receiving syatem; (3) photons that reach the target surface scattered by the aerosol during transmission and eventually return to the receiving system. At this time, the power $P_{E}$ of the laser echo signal can be expressed as:

(2) $$P_{E}=P_{E1}+P_{E2}+P_{E3}$$

where $P_{E1}$, $P_{E2}$, and $P_{E3}$ correspond to the laser echo signal power generated by the above three different types of photons.

(1) For the first case, the photon is directly reflected by the target and then received by the receiving optical system. During the multiple scattering process, the optical path length accumulated, and time delay occurred between the echo signal and the transmitted signal. During the approach of the projectile-target encounter, if the forward speed of the fuze is *v*, and the target is detected for consecutive *k* times, each time interval is $T_{c}$, the delay time $\tau_{1}$ generated by the photon transmitting between fuze and target can be calculated by:
  (3) $$\tau_{1}=\frac{2(R-kT_{c}v)}{c},\;\;k=0,1,2\cdots$$
  where *R* is the distance between fuze and target. If the transmission power is $P_{T_{1}}$, the power of the echo signal formed by the first type photons is:
  (4) $$\begin{array}{c} \begin{array}{c} P_{E1}\left(t\right)=A_{1}\cdot cos\left[2\pi f_{0}\left(t-\tau_{1}\right)+\pi \mu {\left(t-\tau_{1}\right)}^{2}\right] \end{array} \end{array}$$
  (5) $$\begin{array}{c} \begin{array}{c} A_{1}=T_{S}P_{T_{1}}\left[\rho_{T}\frac{e^{-\sigma (2\left(R-kT_{c}v\right))}\cos\theta_{r_{1}}}{{\left(2\left(R-kT_{c}v\right)\right)}^{2}}\right] \end{array} \end{array}$$
  (6) $$T_{S}=D^{2}\eta T_{T}T_{R}$$
  where σ is the extinction coefficient of the atmosphere to the laser, $\theta_{r_{1}}$ is the direction angle when the photon is received, $T_{S}$ is the coefficient related to the optical system characteristics of the fuze, *D* is the diameter of aperture of the receiving optical system, η is the emission system efficiency factor, $T_{T}$ is the laser emission solid angle, and $T_{R}$ is the transmission coefficient of the receiving optical system.
(2) For the second case, received photons don’t reach the target surface and are all scattered by smoke. Assuming that the laser emission power is $P_{T_{2}}$, the photon reaches the surface of the smoke after moving $R_{S}$, and then returns to the fuze after scattering once. According to the single scattering model [20], if the delay time is not considered, the echo signal power obtained by the scattering is:
  (7) $$\begin{array}{c} P_{\delta V}=\int_{V_{2}}T_{S}\left[\frac{\exp\left(-\sigma \left(2R_{S}\right)\right)cos\theta_{r}}{\left(2R_{S}\right)}\right]\times \frac{\gamma_{sca}\exp\left(-\gamma_{ext}\cdot l_{1}\right)}{l_{1}^{2}}P_{T_{2}}dV \end{array}$$
  where $\gamma_{sca}$ is the smoke aerosol scattering coefficient, $\gamma_{ext}$ is the smoke aerosol extinction coefficient, $l_{1}$ is the optical path of photon scattered once, $\theta_{r}$ is the direction angle when the photon is received by receiving optical system, and dV represents the unit space volume at which photons are scattered.
  We construct multiple scattering models based on single scattering model [20,21], the power expression of a photon after *i* times scattering is:
  (8) $$P_{\delta V_{i}}=P_{\delta V_{i-1}}\int_{v_{i}}\frac{\gamma_{sca}\cdot P\left(\theta_{i}\right)\exp\left(-\gamma_{ext}\cdot l_{i}\right)}{l_{i}^{2}}dV_{i}$$
  where $\theta_{i}$ is the direction angle at the *i*-th scattering. According to the Mie scattering theory, the scattering phase function P(θ) in a smoke aerosol environment can be considered to be related only to the photon scattering direction angle, using the Henyey–Greenstein phase function [H-G] expressed [22]:
  (9) $$P\left(\theta \right)=\frac{1-g^{2}}{{\left(1+g^{2}-2g\cos\theta \right)}^{\frac{3}{2}}}$$
  where *g* is asymmetric factor and its value range is [−1,1].
  The delay time $\tau_{2}$ of the photon through $N_{2}$ times of scattering can be calculated by:
  (10) $$\tau_{2}=\frac{2R_{S}-2kT_{c}v}{c}+\sum_{i=1}^{N_{2}}\frac{l_{i}}{c},\;\;k=0,1,2\cdots$$
  The echo signal power composed of the second type of photons after $N_{2}$ times of scattering is:
  (11) $$P_{E2_{N_{2}}}\left(t\right)=A_{2_{\left(i,N_{2}\right)}}\cdot \cos\left\{2\pi \left[f\left(t-\tau_{2}\right)+\frac{1}{2}\mu {\left(t-\tau_{2}\right)}^{2}\right]\right\}$$
  (12) $$A_{2_{\left(i,N_{2}\right)}}=\int_{V_{1}}\ldots \int_{V_{N_{2}}}\begin{array}{c} T_{S}P_{T_{1}}\rho_{s}\gamma_{sca}^{N_{2}}\cos\theta_{r_{N_{2}}}\left[p\left(\theta_{1}\right)p\left(\theta_{2}\right)\cdot \cdot \cdot p\left(\theta_{N_{2}}\right)\right]\\ \times \frac{\exp[-\sigma \left(2\left(R_{S}-kT_{c}v\right)\right)-\gamma_{ext}\sum_{i=1}^{N_{2}}l_{i}]}{{\left(2R_{S}-2kT_{c}v\right)}^{2}l_{1}^{2}l_{2}^{2}\cdot \cdot \cdot l_{N_{2}}^{2}} \end{array}dV_{1}\cdot \cdot \cdot dV_{N_{2}}$$
  where $\rho_{s}$ is the reflection coefficient of aerosol material.
  In the study, it is assumed that there are $M_{2}$ photons scattered by the aerosol into the receiving system; they scattered $N_{2}$ times. The signal formed by the i-th scattered photon can be expressed as $P_{E2_{\left(i,N_{2}\right)}}$, the scattering angle is $\theta_{i,N_{2}}$, the scattering optical path is $l_{i,N_{2}}$, and the direction angle when photon is received is $\theta_{r_{\left(i,N_{2}\right)}}$. The power of the echo signal formed by the first photon can be expressed as:
  (13) $$P_{E2}\left(t\right)=\sum_{i=1}^{M_{2}}P_{E2_{\left(i,N_{2}\right)}}\left(t\right)$$
(3) For the third case, photons that reach the target surface are scattered by the aerosol and eventually return to the fuze receiving system. The scattering process of photons in smoke is consistent with the first case, assuming that the power of the laser-emitting signal is $P_{T3}$. If the photon collides with the target at the *i*-th scattering, the target is the Lambert scatterer, and the reflection coefficient of the target is $\rho_{T}$. The direction angle is $\theta_{T_{i}}$, so the laser power reflected by the target is:
  (14) $$P_{\delta V_{i+1}}=P_{\delta V_{i}}\times \rho_{T}\cos\left(\theta_{T_{i}}\right)$$
  The multiple scattering process caused the optical path length to accumulate and generated time delay. The delay time $\tau_{3}$ of the photon through $N_{3}$ times of scattering can be calculated by:
  (15) $$\tau_{3}=\frac{2R_{S}-2kT_{c}v}{c}+\sum_{i=1}^{N_{3}}\frac{l_{i}}{c},\;\;k=0,1,2\cdots$$
  Therefore, the echo signal power composed of the third type of photons after the $N_{3}$-th scattering can be expressed as:
  (16) $$P_{E3_{N_{3}}}\left(t\right)=A_{3_{\left(i,N_{3}\right)}}\cdot \cos\left\{2\pi \left[f\left(t-\tau_{3}\right)+\frac{1}{2}\mu {\left(t-\tau_{3}\right)}^{2}\right]\right\}$$
  (17) $$A_{3_{\left(i,N_{3}\right)}}=\int_{V_{1}}\ldots \int_{V_{N_{3}}}\begin{array}{c} T_{S}P_{T_{3}}\rho_{T}\cos\theta_{T_{N_{3}}}\rho_{s}\gamma_{sca}^{N_{3}}\cos\theta_{r_{N_{3}}}\left[p\left(\theta_{1}\right)p\left(\theta_{2}\right)\cdot \cdot \cdot p\left(\theta_{N_{3}}\right)\right]\\ \times \frac{\exp[-\sigma \left(2\left(R_{S}-kT_{c}v\right)\right)-\gamma_{ext}\sum_{i=1}^{N_{3}}l_{i}]}{{\left(2R_{S}-2kT_{c}v\right)}^{2}l_{1}^{2}l_{2}^{2}\cdot \cdot \cdot l_{N_{3}}^{2}} \end{array}dV_{1}\cdot \cdot \cdot dV_{N_{3}}$$
  If there are $M_{3}$ photons of the second type, the echo signal power composed of the second type of photons can be expressed as:
  (18) $$P_{E3}\left(t\right)=\sum_{i=1}^{M_{3}}P_{E3_{\left(i,N_{3}\right)}}\left(t\right)$$

If the photoelectric conversion efficiency of the photodetector is $\eta_{e}$, after the photoelectric converter is converted, the received signal can be expressed as:

(19) $$s_{r}\left(t\right)=\eta_{e}\left[P_{E1}\left(t\right)+P_{E2}\left(t\right)+P_{E3}\left(t\right)\right]$$

In this study, the local oscillator signal expression is:

(20) $$s_{LO}\left(t\right)=A_{0}\cos\left[2\pi (f_{0}t+\frac{1}{2}\mu t^{2})\right]$$

According to the principle of FMCW laser detection system, shown in Figure 1, the IF signal of the fuze is obtained by mixing the echo signal with the local oscillator signal. In addition, the random noise of the system is unavoidable. There are various sources of this random noise, including circuit thermal noise, dark current noise, photon shot noise, mixer frequency conversion loss, noise from AD sampling and low pass filtering, and so on. This random noise can be simulated with Gaussian white noise. Therefore, a Gaussian white noise component n(t) is added to the IF signal, and the IF signal expression is:

(21) $$s_{IF}\left(t\right)=s_{r}\left(t\right)\cdot s_{LO}\left(t\right)+n\left(t\right)$$

If the mixing process of the local oscillator signal and the echo signal does not consider the signal attenuation and filters out the higher harmonic term, the IF signal of the FMCW laser fuze in the approach of the projectile can be expressed as:

(22) $$\begin{array}{c} \begin{array}{cc} s_{IF}\left(t\right) & =\frac{\eta_{e}A_{0}}{2}\{A_{1}\cdot \cos\left[2\pi \left(f_{0}+\mu t\right)\left(\frac{2R-2kT_{c}v}{c}\right)\right]\\ \; & +A_{2_{\left(i,N_{2}\right)}}\cdot \cos\left[2\pi \left(f_{0}+\mu t\right)\left(\frac{2R_{S}-2kT_{c}v}{c}+\sum_{j=1}^{N_{2}}\frac{l_{i,j}}{c}\right)\right]\\ \; & +A_{3_{\left(i,N_{3}\right)}}\cdot \cos\left[2\pi \left(f_{0}+\mu t\right)\left(\frac{2R_{S}-2kT_{c}v}{c}+\sum_{j=1}^{N_{3}}\frac{l_{i,j}}{c}\right)\right]\}+n_{m}\left(t\right)\\ \; & =s_{IF1}\left(t\right)+s_{IF2}\left(t\right)+s_{IF3}\left(t\right)+n_{m}\left(t\right) \end{array} \end{array}$$

Under smoke interference conditions, the target echo signal $s_{IF1}\left(t\right)$, the smoke-scattered target echo signal $s_{IF2}\left(t\right)$, the IF signal is a mixture of the smoke backscattered echo signal $s_{IF3}\left(t\right)$, and the random noise. $s_{IF1}\left(t\right)$ is the key signal required for fixed-distance. Ideally, $s_{IF1}\left(t\right)$ is a single frequency cosine signal whose frequency has a linear relationship with the distance between fuze and target.

Visibility is represented by smoke density during simulation. The relationship between smoke density and visibility is as follows [23]:

(23) $$C=\frac{c_{0}}{{v_{x}}^{\gamma }}$$

where $v_{x}$ is the visibility and the unit is m; $c_{0}$ and γ are constants. Typical values for these constants are $c_{0}=37.3$ and γ=1.07 [23,24]. Normally, when the visibility is 10 m, smoke density is 3.2 g/m${}^{3}$, and when the visibility is 50 m, the smoke density is 0.57 g/m${}^{3}$. In Section 4, we set the smoke density to 3.2 g/m${}^{3}$ and aerosol partical diameter to 0.1–100 μm. In Section 5, we used an atmospheric visibility sensor to calibrate the visibility of the experimental environment to 10 m.

## 3. Accurate Fixed-Distance of FMCW Laser Fuze Using a CSD Algorithm

### 3.1. Correntropy Spectral Density Theory

CSD can compare the similarity of two signals in the frequency domain. Therefore, when using CSD algorithm for fixed-distance, computers are used to calculate IF signal frequency under ideal conditions according to the expected distance, and get the signal waveform, that is, the waveform of the reference IF signal. Ideally, an IF signal is a single frequency cosine signal whose frequency has a linear relationship with the distance between fuze and target. The IF signal under ideal conditions is referred to as the reference IF signal. According to the principle of FMCW laser detection, the reference IF signal $s_{IFe}\left(t\right)$ is a cosine signal with a certain frequency $f_{IFe}$. $f_{IFe}$ can be calculated by Equation (24):

(24) $$f_{IFe}=\frac{2B}{T_{m}c}R_{e}$$

where $R_{e}$ is the expected distance.

This paper uses the CSD to compare the similarity in frequency domain between the reference IF signal and the measured IF signal as a criterion for fuze determining distance. If a spectral peak appears at $f_{IFe}$ in correntropy spectral distribution, it means that both signals include this frequency and actual distance from the target is close to the expected distance.

In this paper, the correntropy expression [13,25] of measured IF signal $s_{IF}\left(t\right)$ and reference IF signal $s_{IFe}\left(t\right)$ is:

(25) $$\begin{array}{c} V\left[s_{IF}\left(t\right),s_{IFe}\left(t\right)\right]=E\left[\kappa_{\sigma }\left[s_{IF}\left(t\right),s_{IFe}\left(t\right)\right]\right]\\ =E\left[\frac{1}{\sqrt{2\pi }\sigma }\exp\left[-\frac{{\left|s_{IF}\left(t\right)-s_{IFe}\left(t\right)\right|}^{2}}{2\sigma^{2}}\right]\right] \end{array}$$

where σ is the Gaussian kernel bandwidth. In this study, σ is determined by Silverman’s rule of density estimation [26]:

(26) $$\sigma ={\left(\frac{4a^{5}}{3N_{s}}\right)}^{\frac{1}{5}}$$

where *a* is standard deviation of the sampled signal and $N_{s}$ is the number of samples.

The Mercer kernel function $\kappa_{\sigma }\left(t\right)$ used for correntropy is a normal distribution pattern [25]. When the two input signals $s_{IF}\left(t\right)$ and $s_{IFe}\left(t\right)$ are similar in the time domain, the value of $s\;=\;s_{IF}\left(t\right)-s_{IFe}\left(t\right)$ is around 0, and the amplitude information is preserved. If the difference between the two signals is large, the uncorrelated part of the two signals is rapidly attenuated to zero in the correntropy. The area of attenuation and the rate of attenuation can be controlled by the setting of σ. Therefore, only the similar components in the two signals are retained in the correntropy, and the non-correlated parts are discarded, which can greatly reduce the difficulty of information extraction.

In the implementation of laser fuze engineering, $s_{IF}\left(t\right)$ and $s_{IFe}\left(t\right)$ are discrete digital signals, which can be expressed as ${\left\{s_{IF}\left(n\right)\right\}}_{n=1}^{N_{s}}$ and ${\left\{s_{IFe}\left(n\right)\right\}}_{n=1}^{N_{s}}$, so the correntropy between them can be estimated by the following formula [14]:

(27) $$\hat{V}\left(m\right)=\frac{1}{N-m+1}\sum_{n=m}^{N}\kappa_{\sigma }\left[s_{IF}\left(n\right),s_{IFe}\left(n-m\right)\right]$$

Fourier transform of $\hat{V}\left(m\right)$ can obtain the correntropy spectral density of ${\left\{s_{IF}\left(n\right)\right\}}_{n=1}^{N_{s}}$ and ${\left\{s_{IFe}\left(n\right)\right\}}_{n=1}^{N_{s}}$ to compare the two signals in the frequency domain similarity. The expression is [14]:

(28) $$C\left(f\right)=\sum_{m=-\infty }^{+\infty }\left[\hat{V}\left(m\right)\right]e^{-j2\pi fm}\geq 0$$

Whether the two signals are similar can be discriminated by the presence or absence of a peak at a reference frequency in the correntropy spectral density distribution. When identifying the frequency peaks in the CSD distribution, a rectangular window function such as Equation (29) can be used to intercept data in a specified bandwidth range to determine whether a peak exists:

(29) $$W\left(f\right)=\left\{\begin{array}{cc} 1, & \left(f_{IFe}-\frac{1}{2}\Delta f_{IFe}\right)\leq f\leq \left(f_{IFe}+\frac{1}{2}\Delta f_{IFe}\right)\\ 0, & \mathrm{other} \end{array}\right.$$

where $\Delta f_{IFe}$ is the effective bandwidth near the peak frequency $f_{IFe}$. It should be set according to the fixed-distance accuracy requirement of the FMCW laser fuze. For example, when the fixed-distance error is $\pm \Delta R_{e}$, $\Delta f_{IFe}$ can be calculated by:

(30) $$\Delta f_{IFe}=2\times \frac{2B}{T_{m}c}\Delta R_{e}$$

If the effective bandwidth $\Delta f_{IFe}$ is too wide, the fixed-distance accuracy will be set at a low level, resulting in the position of laser fuze detonation is not ideal. Due to the high speed of the FMCW laser fuze, the signal processing time is very limited, and a narrow effective bandwidth $\Delta f_{IFe}$ may cause the recognition failure of the target signal spectrum peaks.

In the absence of smoke interference, there is only target signal $s_{IF1}\left(n\right)$ and random noise $n_{i}\left(n\right)$ in $s_{IF}\left(n\right)$. At this time, if the frequency of reference IF signal is consistent with the target signal frequency, then $s_{IF1}\left(n\right)$ and $s_{IFe}\left(t\right)$ have only the difference in amplitude and phase. If the delay between $s_{IF1}\left(n\right)$ and $s_{IFe}\left(t\right)$ is $D_{n}$, then there is $s_{IF1}\left(n-D_{n}\right)=s_{IFe}\left(n\right)$. In the expression of $\hat{V}\left(m\right)$, there is $s_{IF}\left(n-D_{n}\right)=s_{IFe}\left(n-m\right)$. When $m=D_{n}$, correntropy has a maximum value. Correspondingly, a distinct peak appears at the frequency $f_{IFe}$ in the correntropy spectral density distribution. At this time, the current distance from target can be determined to satisfy the fixed-distance condition. Under the condition of smoke interference, the target information is hard to extract. In the correntropy, only the similar components in the two signals are retained, and the non-correlated parts are discarded, which can greatly reduce the difficulty of information extraction.

### 3.2. FMCW Laser Fuze Fixed-Distance Processing Using a CSD Algorithm

The basic steps of the CSD-based fixed-distance algorithm are as follows:

(1) Obtain the currently measured IF signal.
(2) Calculate the reference frequency $f_{IFe}$ according to the distance $R_{e}$, and generate a reference IF signal with a frequency $f_{IFe}$.
(3) Calculating $\hat{V}\left(m\right)$ of the currently measured intermediate frequency signal and the reference intermediate frequency signal.
(4) Do Fourier transform for the $\hat{V}\left(m\right)$ to get $C\left(f\right)$.
(5) Determine whether the peak appears in the CSD processing result. If the peak appears at the reference intermediate frequency $f_{IFe}$, step 6) is entered; if there is no peak, the first step is returned to start the detection again.
(6) Recalculate the distance according to the motion speed of the fuze and the detection time interval, and begin the second detection at step 1) until the peak of the continuous measurement is stable, which proves that the target is detected.

Figure 2 shows the process of continuous detection. For the reference IF signal, if the initial value of $R_{e}$ is $R_{1}$, the complete expression of $f_{IFe}$ is:

(31) $$\begin{array}{cc} f_{IFe}=\frac{2B}{T_{m}c}R_{e}=\frac{2B}{T_{m}c}\left[R_{1}-\left(k-1\right)T_{c}v\right] & k=1,2\ldots \ldots \end{array}$$

where *k* is the number of detections, $T_{c}$ is the detection time of the fuze, and *v* is the motion speed of the fuze.

## 4. Simulation

This section studies the CSD algorithm to identify target signals in the case of target without smoke interference, smoke interference but no target, and the target is covered by smoke. The CSD algorithm processing results are compared with the FFT algorithm processing results to further verify the advantages of the CSD algorithm in terms of under smoke interference conditions. The specific parameters used in simulation are shown in Table 1. According to the requirements, the fixed-distance accuracy of the fuze is ±30 cm, so $\Delta f_{IFe}$ is set to 4 KHz calculated by Equation (30). Other parameters setting is consistent with [9]. Table 2 shows theoretical frequency corresponding to 3 m, 5 m, 9 m, and 12 m calculated according to Equation (24).

In this section, when expected distance is 3 m, the simulation results are compared when the distance is 3 m and 5 m between fuze and target. When expected distance is 9 m, the simulation results are compared when the distance is 9 m and 12 m between fuze and target. Each simulation result includes two consecutive detetion, the red curve represents the first detection, and the black curve represents the second detection.

### 4.1. Simulation without Smoke Interference

**Figure 3:**
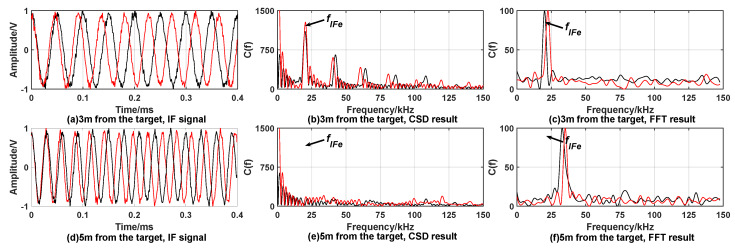
Simulation results when expected distance is 3 m without smoke interference. When fuze is 3 m away from the target, (**a**) is time domain waveform of the IF signal; the processing results of CSD algorithm and FFT algorithm are (**b**,**c**), respectively. When fuze is 5 m away from the target, (**d**) is time domain waveform of IF signal; the processing results of CSD algorithm and FFT algorithm are (**e**,**f**), respectively.

**Figure 4:**
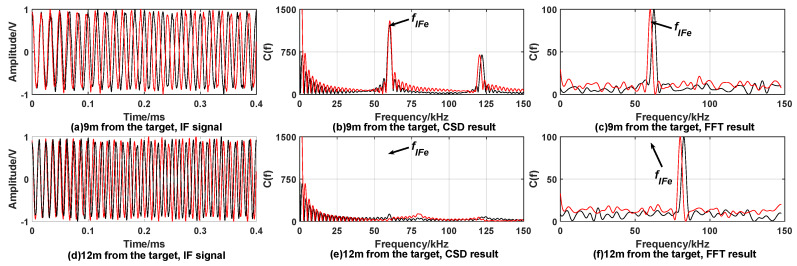
Simulation results when expected distance is 9 m without smoke interference. When fuze is 9 m away from the target, (**a**) is time domain waveform of IF signal; the processing result of CSD algorithm and FFT algorithm are (**b**,**c**), respectively. When fuze is 12 m away from the target, (**d**) is time domain waveform of IF signal; the processing result of CSD algorithm and FFT algorithm are (**e**,**f**), respectively.

Figure 3 and Figure 4 show the simulation results under the condition of no smoke interference.

It can be seen from the simulation results that, when there is no smoke interference, if the distance between the fuze and the target is equal to the expected distance, both the CSD algorithm and the FFT algorithm processing results have obvious peaks at the reference intermediate frequency, both satisfying the fixed-distance condition. However, the amplitude of the spectral peak of the CDS algorithm is much larger than the peak of the FFT algorithm. When the distance between the fuze and the target is not equal to the expected distance, the processing results of the two methods have no peak near the reference intermediate frequency, indicating that the fixed-distance condition is not satisfied. Since the correntropy is always greater than zero, when the correntropy is Fourier transformed to obtain the correntropy spectral density, zero-frequency interference occurs in the result. Zero-frequency interference can be eliminated by the window function without affecting the identification of the peak of the target signal.

### 4.2. Simulation with Smoke Interference but No Target

**Figure 5:**
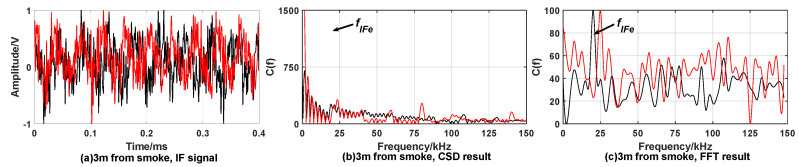
Simulation results when expected distance is 3 m with smoke interference but no target. When fuze is 3 m away from smoke, (**a**) is time domain waveform of the IF signal; (**b**) is the processing result of the CSD algorithm; (**c**) is the processing result of the FFT algorithm.

**Figure 6:**
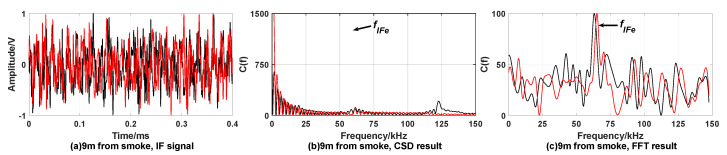
Simulation results when expected distance is 9 m with Smoke interference but no target. When fuze is 9 m away from smoke, (**a**) is time domain waveform of the IF signal; (**b**) is the processing result of CSD algorithm; (**c**) is the processing result of the FFT algorithm.

Figure 5 and Figure 6 show the simulation results under the condition of smoke interference but no target.

When there is no target, it can be found that there is no obvious peak in the result of the CSD algorithm from the results of simulations. However, affected by smoke interference, the result of FFT algorithm has multiple peaks, which can’t achieve fixed-distance and may cause false alarms.

### 4.3. Simulation of Target Being Covered by Smoke

**Figure 7:**
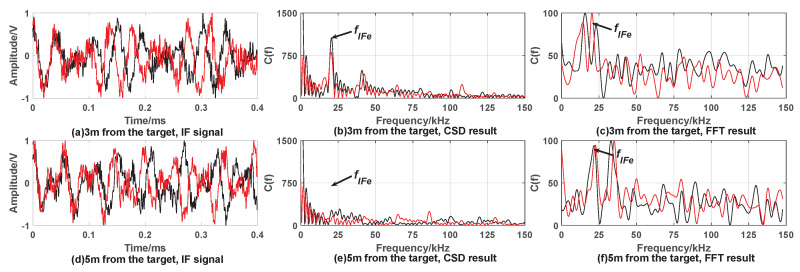
Simulation results when the target is covered by smoke with expected distance 3 m. When fuze is 3 m away from the target, (**a**) is time domain waveform of IF signal; the processing result of CSD algorithm and FFT algorithm are (**b**,**c**), respectively. When fuze is 5 m away from the target, (**d**) is time domain waveform of IF signal; the processing results of CSD algorithm and FFT algorithm are (**e**,**f**), respectively.

**Figure 8:**
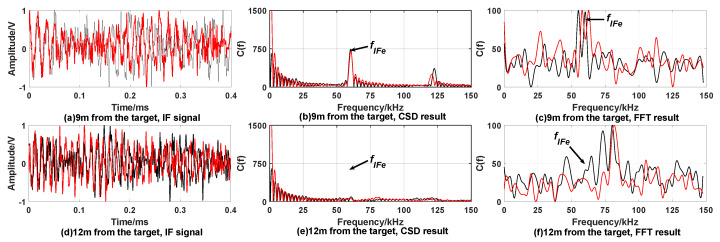
Simulation results when the target is covered by smoke with expected distance 9 m. When fuze is 9 m away from the target, (**a**) is time domain waveform of IF signal; the processing result of CSD algorithm and FFT algorithm are (**b**,**c**), respectively. When fuze is 12 m away from the target, (**d**) is time domain waveform of IF signal; the processing result of CSD algorithm and FFT algorithm are (**e**,**f**), respectively.

Figure 7 and Figure 8 show the simulation results under the condition of target being covered by smoke.

From simulation results Figure 7 and Figure 8, it can be found that the CSD algorithm results in Figure 7b and Figure 8b showing obvious peaks at the reference intermediate frequency, satisfying fixed-distance condition. Figure 7e and Figure 8e show smaller or no peaks. It can be considered to be caused by smoke interference and does not satisfy the fixed-distance condition. The FFT algorithm is greatly affected by smoke interference, and multiple peaks appear. It is impossible to judge whether the distance determination condition satisfies.

In summary, the CSD algorithm can effectively suppress smoke interference. When performing correntropy calculations, the Gaussian kernel function rapidly attenuates the components whose frequency is not equal to $f_{IFe}$ and the target signal with the frequency $f_{IFe}$ is retained, thereby suppressing the smoke interference. The FFT algorithm cannot effectively suppress the interference of smoke.

## 5. Experiment

**Figure 9:**
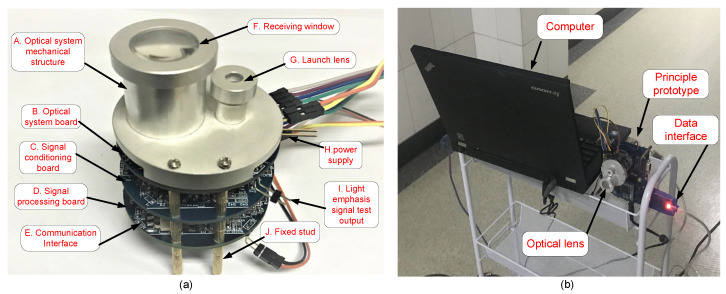
(**a**) shows the composition of FMCW laser fuze prototype; (**b**) the collected IF signal data are transmitted to the computer for processing.

This section builds the FMCW laser fuze test platform, and conducts experiments in different environments. A typical semiconductor laser (λ = 940 nm, 50 mW) was used in the FMCW laser fuze. After being collimated by the optical system, the laser spot size is about 2.5 mm × 1.0 mm, and beam-divergence angle is less than 3 degrees. An avalanche photo-diode detector (APD, AD500-10) was used as the laser detector. In addition, the target in the experiment is a white aluminum plate (1.5 m × 1 m), and its reflectivity is about 80%. The collected IF signal data are transmitted to the computer for processing to verify the anti-interference effect and feasibility of the CSD algorithm. The prototype of the FMCW laser fuze is shown in Figure 9a. Figure 9b show the data processing platform.

In experiments, the fuze is facing the target, and the target surface is perpendicular to the emitted laser beam. The fuze adopts a triangular wave frequency modulation system, and the DDS generates a chirp signal with a bandwidth of 50 MHz, and the modulation period is 100 μs, wherein the up-and-down frequency sweep period is 50 μs that is, $T_{m}=50\;\mu$s. For the convenience of research, the AD sampling frequency is set to 20.48 MHz, the single sampling period is 400 μs, the sampling point is 8196 points, and the cutoff frequency of the IF signal spectrum is 150 kHz.

### 5.1. Performance Test of Fuze Prototype under Conditions of No Smoke Interference

**Figure 10:**
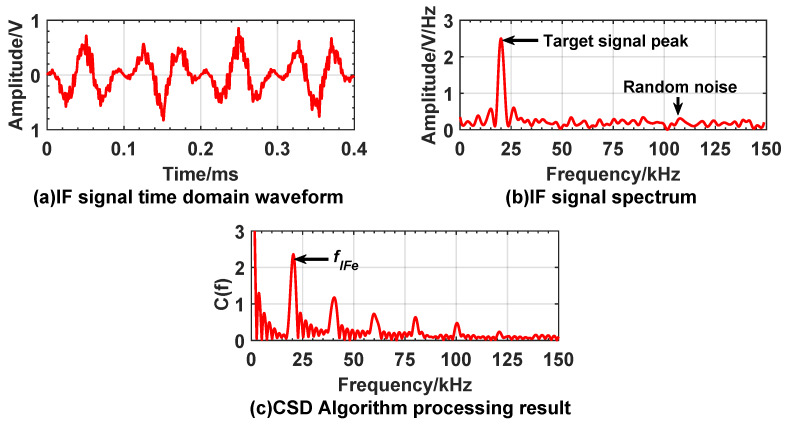
Experiment result when expected distance is 3 m with no smoke interference. (**a**) is time domain waveform of IF signal; (**b**) is spectrum of IF signal; (**c**) is processing result of the CSD algorithm.

**Figure 11:**
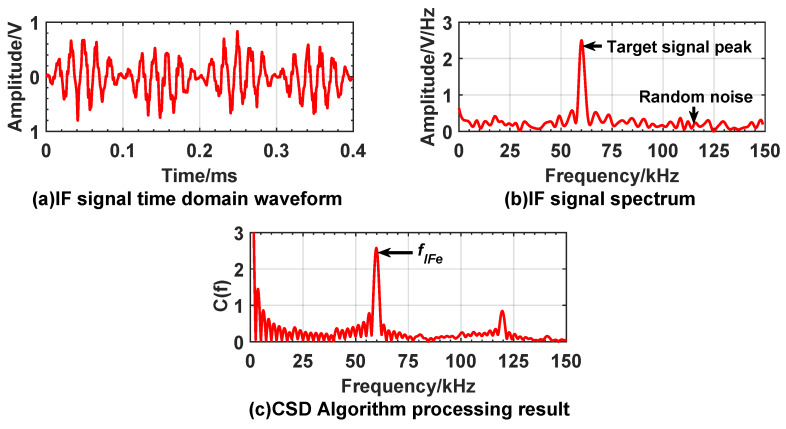
Experiment result when expected distance is 9 m with no smoke interference; (**a**) is time domain waveform of IF signal; (**b**) is spectrum of IF signal; (**c**) is processing results of the CSD algorithm.

Figure 10 and Figure 11 show the experiment results under the condition of no smoke interference, when fuze is 3 m and 9 m away from the target. The time domain waveforms, frequency domain waveforms of IF signal and the processing results using the CSD algorithm are included in the figures.

The spectrum of IF signal is obtained by fast Fourier transform. It can be found that, in the correntropy spectral density distribution, as shown in Figure 10c and Figure 11c, the spectral peak amplitudes at frequencies of 20.48 kHz and 60.15 kHz are significantly higher than those of other frequency peaks, indicating that the correntropy of the measured IF signal and the reference IF signal reaches maximum value. The similarity is the largest. Table 2 shows that the theoretical frequencies corresponding to 3 m and 9 m are 20.01 kHz and 60.04 kHz which are used as the conventional true values. Based on the proportional relationship between the difference value and the conventional true value, the relative error of the frequency does not exceed 5%. The absolute error of distance can also be calculated by Equation (24). The absolute error does not exceed 0.15 m.

### 5.2. Performance Test of Fuze Prototype under Smoke Interference

**Figure 12:**
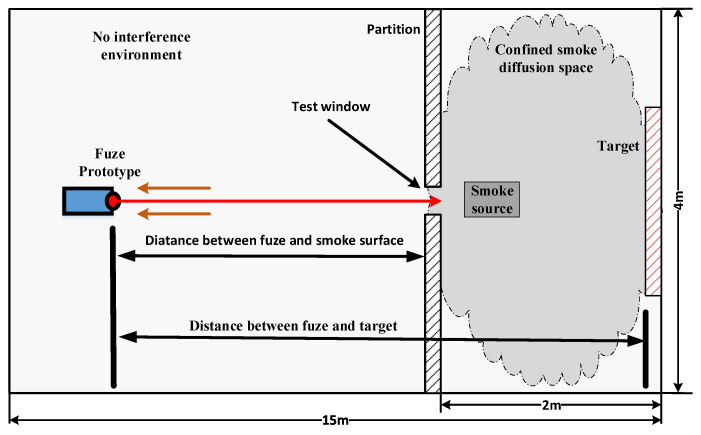
Schematic test scenario of fuze prototype under smoke interference. The smoke interference environment test was conducted in a closed room, and confined diffusion space was isolated by partitions.

This section verifies the anti-interference ability of FMCW laser fuze in the smoke environment. The test scenario schematic is shown in Figure 12.

**Figure 13:**
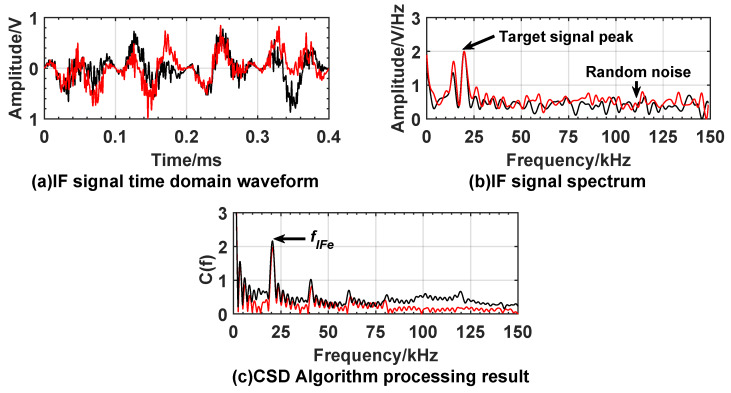
Experiment result when expected distance is 3 m under smoke interference. (**a**) is time domain waveform of IF signal; (**b**) is a spectrum of the IF signal; (**c**) is the processing result of the CSD algorithm. Each simulation result includes two detetions; the red curve represents the first detection, and the black curve represents the second detection.

**Figure 14:**
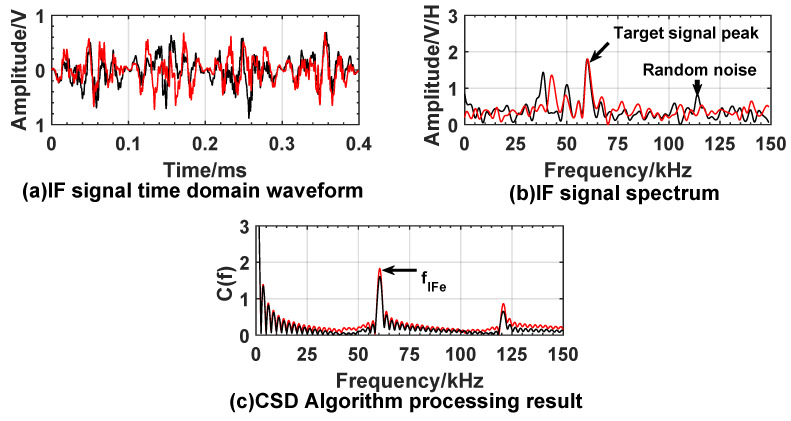
Experiment results when expected distance is 9 m under smoke interference. Figure layout is the same as Figure 13. Each simulation result includes two detections, the red curve represents the first detection, and the black curve represents the second detection.

The experiment room size is 15 m × 4 m wide, test window size is 0.3 m × 0.3 m and confined diffusion space which is 2 m × 4 m wide. Gradually release smoke from smoke source in confined space to reduce visibility. We used an atmospheric visibility sensor to calibrate the visibility of the experimental environment to 10 m. The laser beam emitted by the prototype enters the smoke diffusion space through the test window. Because high speed movement of fuze cannot be simulated indoors, this paper lists the results of two detections of the target under the same conditions. Figure 13 and Figure 14 show the experiment results under the condition of smoke interference, when fuze is 3 m and 9 m away from the target.

From the experimental results, it can be found that the smoke interference increases the noise in the IF signal, and the time domain waveform is obviously distorted. The noise in the spectrum increases, and a high-amplitude interference peak appears on the left of the target signal peak. The smoke interference signal may be used as the target signal to cause false alarm. Under smoke interference conditions, significant peaks can still appear at the reference frequency in the CSD algorithm processing results. The peak frequencies at 3 m and 9 m from the target are 19.6 kHz and 59.5 kHz, respectively. Table 2 shows that the theoretical frequencies corresponding to 3 m and 9 m are 20.01 kHz and 60.04 kHz. Based on the same error analysis in Section 5.1, the relative error between measured peak frequency and theoretical frequency does not exceed 5%, and the absolute error of distance calculated by Equation (24) does not exceed 0.15 m.

## 6. Conclusions

In this paper, a multiple scattering model of photons in smoke environment is established. This model takes account of the multiple scattering of photons and multi-path propagation. The CSD algorithm is used to process the data, and an accurate fixed-distance algorithm of FMCW laser fuze is proposed. The experimental results show that the CSD algorithm has good anti-interference ability in smoke environments, and our experimental results are consistent with the simulation results. The relative error between measured peak frequency and theoretical value does not exceed 5%, and the absolute error of distance does not exceed 0.15 m. Our detection system uses single-quadrant and single-detector achieving high accuracy, so our system is simpler and easier to implement in engineering.

## Figures and Tables

**Table 1:** Simulation parameters.

Parameters	Value
modulation period $T_{m}$	50μs
modulation bandwidth *B*	50 MHz
detection time of fuze $T_{c}$	1 ms
the speed of light *c*	299,792,458 m/s
speed of fuze *v*	200 m/s

**Table 2:** The distance and frequency.

Distance	Frequency
3 m	20.01 kHz
5 m	33.36 kHz
9 m	60.04 kHz
12 m	80.06 kHz
